# A Report of Four Cases of Abscess of the Penis

**Published:** 1875-05

**Authors:** E. T. Easley

**Affiliations:** Dallas, Texas


### A REPORT OF FOUR CASES OF ABSCESS OF THE PENIS.

BY E. T. EASLEY, A. M., M. D., DALLAS, TEXAS.

I am not aware that abscess of the penis is considered a very rare affection, and yet in most systematic works it is dismissed with only cursory notice. It is often enough mentioned as a complication of acute gonorrhœa, and we are told in stereotyped phraseology that incision must be done early, and the pus evacuated. This is all very well as far as it goes, and the danger of temporizing does not need any illustration. But free cutting is not, in all portions of the organ, so simple a thing as the casual allusion to the matter would lead us to believe. It is not difficult to understand how the trabecular structure of the corpora cavernosa might be so imperiled, and the function of erection partially or fully impaired. Those who choose may talk about "laying open the penis," and may do it, but for my part in this particular at least I feel disposed to " touch the bantling tenderly."

The cases I now give, have nothing in them I presume different from what is ordinarily done, and observed in such instances. Considering the comparative variety of the trouble, it is a little singular that they occurred in a private practice, within a period of less than sixty days. Whatever may be the thought of the propriety or success of efforts to discuss bubo, I will state that I have never succeeded in making any such impression on these deposits in the penis.

CASE I.—D. W. T., a merchant aged 27, applied to me. His antecedents were good. The patient presented a very irritable condition of the urethra, with epididymitis, following an acute gonorrhœa. I gathered from his account that the present condition was attributable to the use of a rather noted nostrum, called in the shops " Injection Brou." I considered a stricture imminent. Pending the treatment, the patient applied to the part a rubber band, such as is used on port-monies to confine his dressings. The result was not at all surprising, I was quite prepared to attribute this second mishap also to his bungling surgery, when I discovered considerable induration of and some slight fluctuation in the corpus spongiosum. The much abused member was liberated from the cord, but iodine, oleate of mercury and other remedies utterly failed to bring about resolution. Nothing was left but the knife. An incision was made carefully, in the long axis of the penis, and a quantity of grumous pus evacuated, a good recovery resulting. I will mention, however, in passing, that this young man's troubles were not yet at an end. I was summoned to attend him a few days after opening the abscess, and found him suffering from acute orchitis of the left testicle. This he was inclined to regard as a crowning calamity, and being a fireman supposed it to have been caused by his exertions when on duty a few nights before my visit. The inflammation gave way under warm anodyne fomentations, and active antiphlogistic treatment.

CASE II.—B. S., a mill owner 52 years old, of irregular habits, and former syphilitic history. Applied to me for treatment with chanchroids around the corona glandis. The complication of abscess made itself known on the dorsum of the organ in a short time. On account of the advanced age and broken-down health of the patient, I was not a little apprehensive of phagædena; my fears fortunately were not realized. The bistoury was again in demand. The patient was ordered freely potassio-tartrate of iron both locally and internally, and made a good recovery. The more I see of the action of this excellent remedy, the more am I convinced of the truth of Ricord's observation concerning it,—" it is the born enemy of phagædena."

Case III.—T. W., performer at a variety theatre, aged 31. On examination after getting the history of this case, I discovered that an abscess had formed and made an opening for itself into the urethra only a little deeper than the fossa navicularis. The man was given an injection of carbolic acid (grs. xv., ℥i,) and directed to use it three or four times daily, compressing the urethra behind the sinus. Pus ceased to flow, and the canal leading from the abscess closed in six days. This case also arose as a complication of acute gonorrhœa. The patient is an athlete, and alleges that his rising dates from an evening when he exerted himself unusually on the trapeze.

Case IV.—G. L., German, clerk, aged 23. Gives no history of recent gonorrhœa or other venereal troubles. Says the abscess which he exhibited was brought on by bruising the member whilst riding a rough trotting horse. Whatever may be thought of his story, (and I do not think it unlikely,) there were no evidences of other disease at the time he presented himself. The collection was cautiously opened, the wound healing kindly.

